# From active escapism to virtual withdrawal: Validation of the Compensatory-Dissociative Online Gaming scales (C-DOGs)

**DOI:** 10.1556/2006.2024.00059

**Published:** 2024-11-26

**Authors:** Alessandro Giardina, Loïs Fournier, Vladan Starcevic, Daniel L. King, Maria Di Blasi, Adriano Schimmenti, Joël Billieux

**Affiliations:** 1 Institute of Psychology, University of Lausanne, Lausanne, Switzerland; 2 Faculty of Medicine and Health, Sydney Medical School, Nepean Clinical School, Brain and Mind Centre, University of Sydney, Sydney, Australia; 3 College of Education, Psychology, & Social Work, Flinders University, Adelaide, Australia; 4 Department of Psychology, Educational Sciences and Human Movement, University of Palermo, Palermo, Italy; 5 Department of Human and Social Sciences, UKE – Kore University of Enna, Enna, Italy; 6 Centre for Excessive Gambling, Addiction Medicine, Lausanne University Hospitals, Lausanne, Switzerland; 7 Department of Behavioral and Cognitive Sciences, University of Luxembourg, Esch-sur-Alzette, Luxembourg

**Keywords:** dissociation, escapism, gaming disorder, measurement, validation

## Abstract

**Background:**

In the Compensatory-Dissociative Online Gaming (C-DOG; Giardina et al., 2024) model, we proposed a continuum from compensatory to dissociative gaming involvement. This continuum represents different degrees of integration between physical and virtual environments with three core processes – Active Escapism, Escape, and Dissociation – and two peripheral processes – Gaming-Related Relaxation and Body-Mind Detachment. Here, we developed and tested a multidimensional measure based on this model.

**Method:**

We capitalized on existing items for measuring escapism and dissociation and we generated new items consistent with the hypothesized model dimensions. A total of 54 items were administered to 1,176 online gamers playing different game genres, together with measures of problematic gaming, passion for gaming, and other psychological distress indicators.

**Results:**

Exploratory and confirmatory factor analyses yielded a six-factor, 36-item structure, with multiple hierarchical regression analyses highlighting unique associations with other psychological constructs assessed.

**Discussion:**

The following factors were identified: (1) *Emotional Displacement* - redirection of negative emotion into the game with associated relaxation; (2) *Absorption* - detachment of the player from time and space while gaming; (3) *Active Escapism* - simulative use of the game to compensate for lack of self-confidence in reaching physical life objectives; (4) *Virtual Withdrawal* – maladaptive gaming to balance impaired social functioning, predicted by traumatic experiences and pervasive depression; (5) *Dissociative Regulation* - dysfunctional level of engagement associated with excessive anxiety; (6) *Failure Escape* - problematic avoidance via gaming related to fear of future failures.

**Conclusions:**

The C-DOG factors identify critical psychological processes associated with problematic gaming, with relevant research and clinical implications.

## Introduction

The concept of escapism in the context of problematic online gaming has been considered of central importance (Blasi et al., 2019; Boldi, Rapp, & Tirassa, 2022; Demetrovics et al., 2011; Guglielmucci et al., 2019; Kardefelt-Winther, 2014; Király et al., 2022; Kosa & Uysal, 2020; Melodia, Canale, & Griffiths, 2022; Pal & Arpnikanondt, 2023; Yee, s.d.). Yet, it is generally agreed that this construct lacks conceptual clarity, generating diverse and inconsistent results that have made comparisons between studies difficult (Hussain, Jabarkhail, Cunningham, & Madsen, 2021). Acknowledging such difficulty, we recently conducted a critical review of compensatory and dissociative mechanisms associated with problematic gaming – including escapism – and proposed the Compensatory-Dissociative Online Gaming (C-DOG) model (Giardina et al., 2024). In this work, we reviewed the most used frameworks and measures of escapism, identifying their strengths and limitations (Demetrovics et al., 2011; Hagström & Kaldo, 2014; Király et al., 2022; Stenseng, Falch-Madsen, & Hygen, 2021; Yee, 2006). The interchangeable use of the concepts of escapism, immersion, and avoidance (i.e., escape), which we defined as the “escapism vicious cycle,” was identified as the main problem preventing the discourse about escapism from moving forward. In our proposal, the first consideration to break this cycle was to focus on the nature of escapism as a psychic movement from the physical to the virtual environment of gaming (Calleja, 2010; Giardina et al., 2023). A psychic movement can be defined as an affective, cognitive, and motivational tension that may or may not concretize into an observable behavior (Orăşanu, 2012). In the case of playing video games, the movement remains primarily psychological, as the immersion in a videogame, being a virtual environment, occurs primarily with the mind rather than with the body (Giardina et al., 2024).

Adopting the metaphor of fluid migrations (Calleja, 2010; Coulson, Oskis, Spencer, & Gould, 2020), we thus delineated a qualitative difference between the psychic movements of escapism and escape (i.e., avoidance), based on their directionality from the physical to the virtual environment (bidirectional vs. unidirectional), duration of the psychological “stay” in the virtual environment (short-lived or temporary vs. enduring) and perception of the situation in the physical environment from which the movement originated (amendable vs. intolerable and rejected). Put differently, the nature of escapism would be that of psychologically - and temporarily - leaving the original environment (the physical one) with the intention to return to and amend it after immersing oneself in a more favorable and resourceful environment (the virtual environment of gaming). On the other hand, the escape movement implies a rejection of the physical environment, leading to a need to leave it more definitively and regardless of where one ends up (Calleja, 2010; Giardina et al., 2023).

In accordance with this first point, our second consideration questioned whether, from a conceptual point of view, escapism should belong to the framework of the motivations to play. In fact, in classical gaming motives models (Demetrovics et al., 2011; Király et al., 2022; Yee, s.d.), escapism is the only motivation that intrinsically implies a discomfort experienced outside the game. We thus proposed a switch to the view that the escapism movement arises from the unsatisfaction of physical world needs (e.g., socialization, achievement, exploration; Giardina et al., 2024; Pal & Arpnikanondt, 2023). That is, when a compensatory process is active (Boldi et al., 2022). Therefore, whenever a specific psychological need is unmet outside of the game, the respective motivation to play should be framed within a broader escapist movement. For example, being motivated by mastering the game should be considered an escapist movement if psychological needs for competence are unmet outside the game. Framed this way, escapism should be conceptualized as a higher-order mechanism in comparison to motivations to play and the key measure of the compensatory process^1^ (Blasi et al., 2019; Boldi et al., 2022; Giardina et al., 2021; Pal & Arpnikanondt, 2023). This constitutes another core distinction of escapism from nonspecific immersion in gaming (i.e., playing the game) or from avoidance/escape.

### The C-DOG model and the current study

**Fig. 1.**
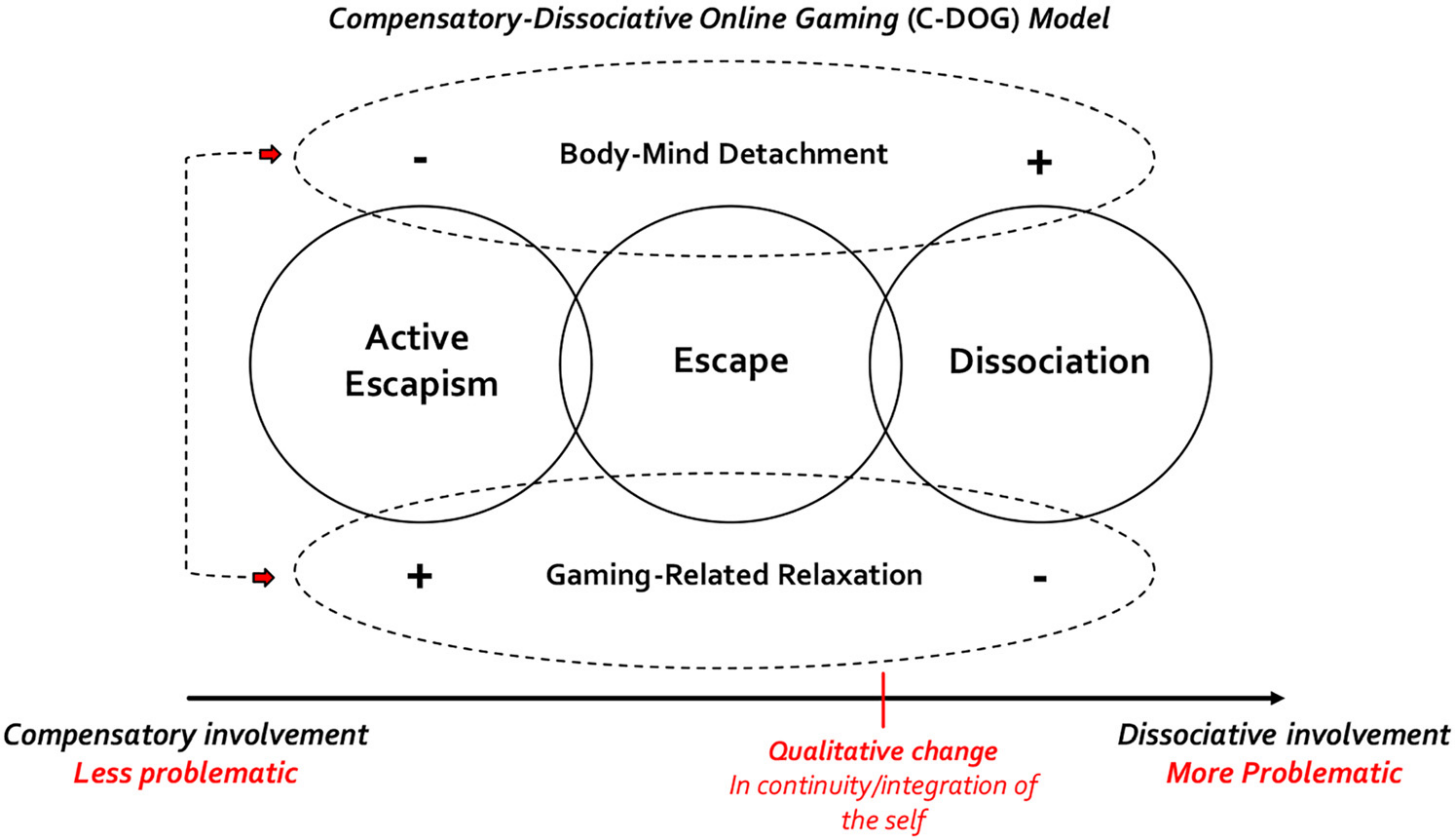
Compensatory-Dissociative Online Gaming (C-DOG) model (Giardina et al., 2024).

Overall, considering escapism as the key measure of the compensatory process in gaming bears the potential to make this construct more specific and allow research and clinical discourse to move forward. In this regard, we posited that virtual gaming environments should no longer be considered in opposition to the “real” environments but rather to the “physical” ones. They would thus be *nonphysical* spaces situated in the everyday reality of many individuals (Giardina et al., 2024; Meriläinen, 2022; Razum & Huić, 2023). We proposed that this feature fosters potential for the simulation of emotional experiences in the game, which could make videogames akin to dreams in the way that they help individuals process their emotions. Such emotional processing could take place by testing potential challenges in satisfying basic psychological needs (*experimental value*) or by anticipating one's potential emotional reactions to different scenarios (*prospective value*; Bown & Gackenbach, 2016; Boyes & Gackenbach, 2016; Domhoff, 2011; Domhoff & Schneider, 2018; Giardina et al., 2024; Meriläinen, 2022; Revonsuo, 2000). Owing to this view, we linked the compensatory and dissociative processes associated with problematic gaming via the C-DOG model (Fig. 1).

The C-DOG model posits a continuum from compensatory (less problematic) to dissociative (more problematic) involvement in online games, representing different degrees of integration between virtual and physical environments with the respective parts of individuals' selves (Giardina et al., 2024; Szolin, Kuss, Nuyens, & Griffiths, 2022), via three core processes. Active Escapism is the key measure of the compensation process and was defined as taking advantage of the simulative environment of gaming to compensate for struggles in satisfying psychological needs. Active Escapism constitutes a bidirectional movement toward the virtual environment, with the intention to return to the physical one to amend or ameliorate it (Boldi et al., 2022; Calleja, 2010; Giardina et al., 2023, 2024; Kuo, Lutz, & Hiler, 2016). Escape represents an intermediate position in the continuum, characterized by rejection and avoidance of the physical world, pushing to seek refuge in the game. Unlike Active Escapism, Escape is a unidirectional movement arising from the rejection of the physical world with little regard for where one ends up (Demetrovics et al., 2011; Giardina et al., 2023; Hagström & Kaldo, 2014; Melodia et al., 2022). Finally, Dissociation represents the disconnection between the two environments and respective parts of the self (Casale, Musicò, Gualtieri, & Fioravanti, 2023; Giardina et al., 2024; Guglielmucci et al., 2019; Schimmenti & Caretti, 2010). We also posited two transversal and interrelated processes: Gaming-Related Relaxation and Body-Mind Detachment. The first refers to a sense of peace and release of tension during gaming, whereas the second has been defined as a mild and physiological disconnection between mind and body arising during access to the virtual world (Larche, Tran, Kruger, Dhaliwal, & Dixon, 2021; Ortiz de Gortari & Griffiths, 2016; Snodgrass, Lacy, Francois Dengah, Fagan, & Most, 2011). In the current study, we aimed to (1) develop and test a self-reported measurement instrument based on the five-factor structure hypothesized by the C-DOG model, (2) identify the main predictors of the C-DOG dimensions, and (3) explore how the C-DOG scales (C-DOGs) contribute to predicting problematic gaming and passion for gaming (i.e., intensive but not problematic pattern of gaming; see, e.g., Billieux, Flayelle, Rumpf, & Stein, 2019; Razum & Huić, 2023). This new scale will open new lines of research in the field and help clinicians with the assessment and treatment of problematic gaming and gaming disorder.

## Method

### Scale development: item generation

The C-DOGs were developed according to the guidelines for scale development proposed by Boateng, Neilands, Frongillo, Melgar-Quiñonez, and Young (2018). Given the extensive literature on the topics of compensation, escapism, and dissociation in gaming, we first capitalized on existing scales to collect or adapt 24 items that could fit the proposed theoretical definitions (see “C-DOGs Items Ref Table Short” available from the Open Science Network [OSF]: https://osf.io/vgrwk/). In the second step of scale creation, we relied on our clinical expertise and the existing literature to generate 30 items representing the hypothesized domains. Among them, 16 were inspired by existing qualitative studies or theoretical frameworks that have not yet been operationalized, and 16 items were entirely generated from our understanding of the hypothesized constructs. A table with the full pool of 54 items organized by dimension and their original frameworks is available from the OSF at https://osf.io/vgrwk/.

### Scale development: translation and content validity

The first version of the scale was created in English, consistent with items retrieved from the existing literature. Subsequently, the items were translated into Italian and then back-translated into English by a professional bilingual English translator who has been living in Italy for over 15 years. This procedure allowed us to capitalize on our English- and Italian-speaking networks for data collection. To ensure the content validity of each specific item and the clarity of its formulation, we performed several rounds of consensus, considering the specific risks identified when creating scales about gaming (King, Haagsma, Delfabbro, Gradisar, & Griffiths, 2013, 2020). To ensure the clarity of the items from the end-users’ perspective, we recruited 20 gamers from a Facebook group dedicated to the game World of Warcraft, who filled out a pretest survey containing the 54 items on a 5-point Likert scale (1 = “not at all representative”; 5 = “fully representative”) with an additional response option in the form of an open answer. To ensure the content validity of each dimension from the expert perspective, the first author provided some coauthors with the definitions of the five theoretical constructs and a list of the full pool of items randomly distributed with the following indications: “*Using different colors, please highlight each item to indicate relaxation, body-mind detachment, active escapism, escape, or dissociation.*” The colored items in the list of each coauthor were subsequently reorganized according to the original dimension hypothesized by the first author. The visual effect of this method facilitated the identification of discrepancies in the understanding of each dimension between authors, allowing them to reallocate or remove items if needed, thus increasing a shared understanding.

### Sampling procedures

Participants were recruited via an online survey of about 40 minutes administered using Qualtrics. The survey was posted on Facebook groups of different videogame genres, such as multiplayer online battle arena (MOBA), battle royale (BR), massive multiplayer online role-playing games (MMORPGs), first-person shooter (FPS), real-time strategy (RTS), and sport games. If willing to leave their e-mail address, players had a 1 in 100 chance to win an Amazon coupon of 20 euros and one additional chance to win a final lottery for a one-year subscription to a service of their choice (e.g., Netflix, PlayStation Plus, Xbox Live), valued at around 100 euros. Data collection started on February 14, 2020, and ended on April 3, 2020, which was about the time frame when the COVID-19 pandemic started in Europe. The survey comprised sociodemographic information (age, sex, education level), gaming-related habits (time spent on gaming per week, online game genre preferences, use of fixed vs. mobile gaming devices, social gaming habits, compensatory use of the game, and degree of consistency between behaviors inside and outside the game), gaming-related questionnaires (distinguishing between passion for gaming and problematic gaming), the C-DOGs full pool of 54 items, and measures of psychological well-being (depression and anxiety, emotion dysregulation, trauma).

### Participants

The minimum age for participating in this study was 18 years old. English-speaking participants were excluded from the sample due to their small number at the end of recruitment. Thus, the final sample consisted of 1,176 Italian-speaking online gamers, predominantly males (87.9%, *n* = 1,034) aged between 18 and 58 (*M* = 25.39; *SD* = 7.12). The most frequent level of education achieved in the sample was a high school degree (63.6%, *n* = 748), followed by a middle school degree (20.7%, *n* = 243) and a bachelor/master's degree (15%, *n* = 176). Concerning gaming habits, participants reported playing on fixed devices (e.g., PCs or consoles; 96.3%, *n* = 1,133) and participating in multiple online game genres such as FPS (e.g., “Rainbow Six Siege”; 65.7%, *n* = 773), BR (e.g., “Fortnite”; 46.9%, *n* = 551), MMORPGs (e.g., “World of Warcraft”; 42.9%, *n* = 504), Sport games (e.g., “Fifa Ultimate Team”; 31.6%, *n* = 372), MOBA (e.g., “League of Legends”; 14.2%, *n* = 167), and RTS (e.g., “Starcraft”; 9.9%, *n* = 116), for a mean of 20.49 hours per week (range = 1–135, *SD* = 15.65). About half of the sample reported being part of a community of gamers (i.e., team, guild) rather than being a lone/casual company player (54.6%, *n* = 642), and most gamers reported meeting people they play online with in physical settings as well (82.9%, *n* = 975). Finally, most of the players reported that their behaviors and interests inside the game were somewhat consistent with those outside the game (43%, *n* = 506), with only a minority of players reporting compensation via gaming a lot or to a full extent for deficiencies in their offline lives (12.6%, *n* = 149). Of note, part of this sample (*n* = 664) was used in the context of a study about the effect of online gaming on self-isolation due to the COVID-19 pandemic (Giardina et al., 2021).

### Measures

The Italian adaptation of the 10-item **Internet Gaming Disorder Test** (IGDT-10; Király et al., 2017) was used to measure problematic gaming. This measure operationalizes problematic gaming according to the *DSM-5* (e.g., “In the past 12 months, have you ever unsuccessfully tried to reduce the time spent on gaming?”; “When you were not playing, how often have you fantasized about gaming, thought of previous gaming sessions, and/or anticipated the next game?”). Participants answered on a 3-point Likert scale from 0 (“never”) to 2 (“often”). Responses were summed, with higher scores representing higher levels of problematic gaming. Confirmatory factor analyses performed on the 10-item Italian adaptation of the measure resulted in a one-factor solution with adequate fit indices (comparative fit index [CFI] = 0.94, Tucker-Lewis index [TLI] = 0.92, root mean square error of approximation [RMSEA] = 0.06, *p* < 0.001). Furthermore, in the current study, we obtained an acceptable alpha of 0.75, which aligns with the results obtained by this scale in its cross-cultural validation (Király et al., 2019).

An Italian adaptation of the ethnographically validated **Videogames Involvement Scale** (VIS; Snodgrass et al., 2017) translated for the purpose of the current study was used to measure high engagement in and passion for gaming (i.e., high but healthy involvement; Billieux et al., 2019). This scale was translated into Italian and back-translated to English by the same bilingual translator of the C-DOGs. The scale includes three questions for each dimension of Yee's (2006) tripartite framework of motivations to play, that is, achievement (e.g., “I feel committed to improving my play, striving to be the best player I can be”), socialization (e.g., “I feel committed to helping online gaming friends have fun and meet their goals”), and immersion (e.g., “I find that gaming can help me to forget about offline concerns”), with additional questions covering general gaming involvement (e.g., “I feel that gaming is a way of life and not just recreation”) and engagement (e.g., “I regularly continue playing even when tired”). In this study, the three items covering immersion were omitted to avoid conceptual overlap and statistical collinearity with the items of the C-DOGs, in accordance with the procedures deployed by Snodgrass et al. (2018). The final version thus consisted of 12 items. Responses on this scale were summed and scored on a 5-point Likert scale from 1 (“strongly disagree”) to 5 (“strongly agree”), with higher scores indicating stronger commitment and passion for gaming. Confirmatory factor analyses performed on the 12-item Italian adaptation of the measure resulted in a one-factor solution with sub-adequate fit indices (CFI = 0.90, TLI = 0.88, RMSEA = 0.10, *p* < 0.001). The adapted VIS in this study showed a Cronbach's alpha of 0.83.

The Italian version of the **Depression Anxiety Stress Scale-21** (DASS-21 (Bottesi et al., 2015; Lovibond & Lovibond, 1995); was used to assess emotional distress. The measure, consisting of a 4-point Likert scale ranging from 0 (“did not apply to me at all”) to 3 (“applied to me very much or most of the time”), evaluated three dimensions: Depression (e.g., “I couldn't seem to experience any positive feeling at all”), Anxiety (e.g., “I experienced breathing difficulty (e.g., excessively rapid breathing, breathlessness in the absence of physical exertion”), and Stress (e.g., “I found it difficult to relax”), with higher scores indicating heightened emotional distress. Internal consistency in the current study reflected previous research findings (*α* = 0.90 for Depression, 0.81 for Anxiety, and 0.87 for Stress; Loton, Borkoles, Lubman, & Polman, 2016).

The 18-item version of the **Difficulties in Emotion Regulation Scale** (DERS) by Victor and Klonsky (2016) was implemented to measure emotion dysregulation. Considering that the Italian validation of the 18-item version of this scale was not published at the time that the survey began (Rossi, Panzeri, & Mannarini, 2023), the Italian version of this short scale was assembled by selecting the translated items of interest from the Italian validation of the full scale (Sighinolfi, Norcini Pala, Chiri, Marchetti, & Sica, 2010). Furthermore, we followed the indications by Hallion, Steinman, Tolin, and Diefenbach (2018) and removed the three reverse items composing the Awareness subscale. Therefore, the final measure was composed of 15 items. Items are rated on a scale from 1 (“almost never”) to 5 (“almost always”). This 15-item version thus included five subscales: lack of emotional clarity (Clarity; e.g., “I am confused about how I feel;” Cronbach's *α* = 0.84), lack of acceptance of one's emotions when distressed (Nonacceptance; e.g., “When I am upset, I feel ashamed with myself for feeling that way;” Cronbach's *α* = 0.83), lack of ability to engage in goal-directed cognition and behavior when distressed (Goals; e.g., “When I am upset, I have difficulty focusing on other things;” Cronbach's *α* = 0.83), lack of ability to manage one's impulses when distressed (Impulse; e.g., “When I am upset, I lose control of my behavior;” Cronbach's *α* = 0.87), and perception of a lack of effective strategies to feel better when distressed (Strategies; e.g., “When I am upset, I believe that I will remain that way for a long time;” Cronbach's *α* = 0.82). Cronbach's alphas for the subscales in this study were similar to those found in other studies (Estévez, Jáuregui, Sánchez-Marcos, López-González, & Griffiths, 2017).

Finally, we implemented two items that evaluated the presence of past **psychological trauma** and its perceived effect on the present. We decided to include this measure based on the consistent link between exposure to psychological trauma and dissociation and experiences of psychological trauma found in the literature (Schimmenti, 2018). The complete list of measures is available from the OSF at https://osf.io/vgrwk/.

### Data analytic strategy

Exploratory and confirmatory factor analyses on the full pool of 54 candidate items were performed to establish the factorial structure of the C-DOGs using *R* version 4.4.0 (R Core Team, 2024). The corresponding 1,176 observations were first semi-randomly split into two subsamples of equal size under the sole constraint of comprising an equal proportion of participants who completed the online survey under COVID-19-related lockdown conditions (*n* = 366) and participants who did not (*n* = 810).

Subsequently, exploratory factor analyses were performed using the *R* packages EFAtools version 0.4.4 (Steiner & Grieder, 2023) and *lavaan* version 0.6–18 (Rosseel, Jorgensen, & De Wilde, 2024) on the full pool of items. With respect to the first subsample (*n*_1_ = 588), the following three-step iterative procedure was conducted:

1. Parallel analyses were performed to evaluate the adequate number of factors to extract (Crawford et al., 2010; Horn, 1965).
2. Oblique rotation and weighted least squares mean-and-variance-adjusted robust estimation methods were used to fit the exploratory structural equation models (DiStefano & Morgan, 2014; Flora & Curran, 2004).
3. Factor loadings (i.e., the presence of low-magnitude factor loadings and “cross-loadings”) were examined to assess the adequacy of the fitted exploratory structural equation model (Kline, 2023a, 2023c).

If all observed variables presented one and only one factor loading (*λ* ≥ 0.400), the fitted exploratory structural equation model was considered for subsequent confirmatory factor analyses. Observed variables not meeting this decision rule were omitted, and a new iteration of the three-step procedure was conducted.

Following exploratory factor analyses, confirmatory factor analyses were performed on the theory-driven and data-driven factor models – derived from the C-DOG theoretical model and the exploratory factor analyses – of the C-DOGs using *R* packages *lavaan* version 0.6–18 (Rosseel et al., 2024) and *semTools* version 0.5–6 (Jorgensen, Pornprasertmanit, Schoemann, & Rosseel, 2022). With respect to the second subsample (*n*_2_ = 588), the following two-step iterative procedure was conducted:

1. Weighted least squares mean-and-variance-adjusted robust estimation methods were used to fit the confirmatory structural equation models (DiStefano & Morgan, 2014; Flora & Curran, 2004).
2. Factor loadings (i.e., the presence of low-magnitude factor loadings) and indicators/factor pragmatics (i.e., the presence of a low number of indicators per factor) were examined to assess the adequacy of the fitted confirmatory structural equation model (Kline, 2023a, 2023c).

If all observed variables presented factor loadings (*λ* ≥ 0.500) and all latent variables presented three or more factor loadings (*λ* ≥ 0.500), the fitted confirmatory structural equation model was considered for the C-DOGs. Observed variables not meeting these decision rules were omitted, and a new iteration of the two-step procedure was conducted.

To evaluate the quality of the adjustment of the theory-driven and data-driven fitted confirmatory structural equation models to the data, we used three conventional fit indices: the CFI, the TLI, and the RMSEA (Kline, 2023b, 2023d). Adequate adjustment to the data was determined by a CFI ≥ 0.900, a TLI ≥ 0.900, and an RMSEA ≤ 0.080 (Browne & Cudeck, 1992; Chen, Curran, Bollen, Kirby, & Paxton, 2008; Kenny, Kaniskan, & McCoach, 2015; Marsh, Hau, & Grayson, 2005; Schermelleh-Engel, Moosbrugger, & Müller, 2003). To evaluate the internal consistency reliability of the theory-driven and data-driven fitted confirmatory structural equation models, we used two conventional internal consistency coefficients: Cronbach's *α* (Cronbach, 1951) and McDonald's *ω* (McDonald, 1999).

After the factorial structure had been established, inter-factor and external correlations were run to ensure the construct validity of the C-DOGs using *SPSS* version 29.0 (IBM Corporation, 2022). Furthermore, different sets of hierarchical multiple regressions were computed to investigate the predictors of the C-DOGs and how the C-DOGs contribute to predicting problematic gaming and passion for gaming. For the first step, we ran six forced entry multiple regression models with each C-DOG factor as the outcome variable, and external correlates (such as emotion dysregulation, emotional distress, and psychological trauma), sociodemographic information (age, sex, education level), and gaming habits (hours per week of gaming, gaming genre played, social vs. nonsocial gaming, compensatory attitude toward gaming, and in-out of the game behavioral consistency) as predictors. Furthermore, we decided to include problematic gaming and passion for gaming as covariates in these models. Subsequently, we entered the significant predictors of the forced entry step into a further forward hierarchical regression, keeping the correspondent C-DOG scale as the outcome variable. The same procedure was performed to investigate the predictive validity of the C-DOG scales, that is, how they contributed to predicting passionate involvement in gaming and problematic gaming. In these regression models, we decided to include online game genres as covariates given the important role they demonstrated to play in problematic gaming (Rehbein, King, Staudt, Hayer, & Rumpf, 2021). In the Results section, we present the predictors of each dimension in the same order that they were introduced into the respective hierarchical models. All outputs, including additional and post hoc analyses, are available from the OSF: https://osf.io/vgrwk/.

### Ethics

The present study has been conducted in accordance with the Declaration of Helsinki and all guidelines for experimental investigation with human subjects required by the University of Luxembourg. At the time of the study, the first and last authors were still working at the University of Luxembourg, and the study design was elaborated and the data collection organized before these authors moved to the University of Lausanne. Participants gave online consent prior to starting the online survey. Participation was voluntary, and the anonymity of the participants was guaranteed.

## Results

**Fig. 2.**
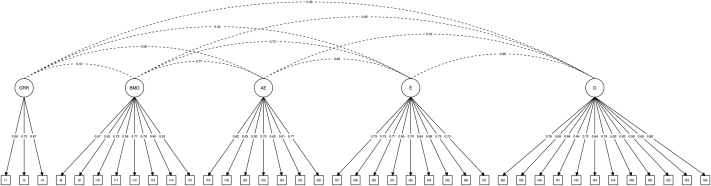
Theory-driven confirmatory structural equation model Circles denote latent variables (i.e., factors). Squares denote observed variables (i.e., items). Single-headed arrows connecting latent variables to observed variables denote model-implied non-null λ standardized estimates (i.e., factor loadings). Dashed lines connecting latent variables denote model-implied non-null φ standardized estimates (i.e., factor covariances). GRR = Gaming-Related Relaxation; BMD = Body-Mind Detachment; AE = Active Escapism; E = Escape; D = Dissociation

**Fig. 3.**
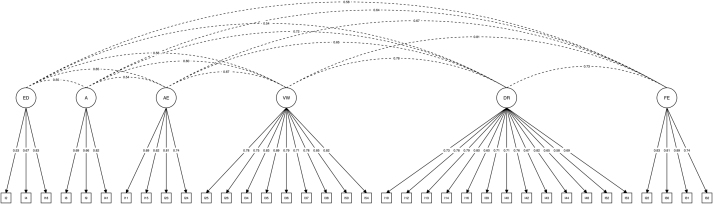
Data-driven confirmatory structural equation model Circles denote latent variables (i.e., factors). Squares denote observed variables (i.e., items). Single-headed arrows connecting latent variables to observed variables denote model-implied non-null λ standardized estimates (i.e., factor loadings). Dashed lines connecting latent variables denote model-implied non-null φ standardized estimates (i.e., factor covariances). ED = Emotional Displacement; A = Absorption; AE = Active Escapism; VW = Virtual Withdrawal; DR = Dissociative Regulation; FE = Failure Escape

Exploratory factor analyses performed on the full pool of items with respect to the first subsample yielded a seven-factor, 40-item fitted exploratory structural equation model after two iterations of the three-step procedure. In the first iteration, parallel analyses suggested that seven factors be extracted, with 42 items presenting one and only one factor loading (*λ* ≥ 0.400). In the second iteration, parallel analyses suggested that seven factors be extracted, with 40 items presenting one and only one factor loading (*λ* ≥ 0.400). A supplementary iteration indicated that the seven-factor, 40-item fitted exploratory structural equation model presented adequate factor loadings. Confirmatory factor analyses performed on the theory-driven factor model of the C-DOGs with respect to the second subsample yielded a five-factor, 39-item fitted confirmatory structural equation model after two iterations of the two-step procedure. In the first iteration, the five-factor, 40-item fitted confirmatory structural equation model presented one item with a factor loading (*λ* < 0.500). In the second iteration, the five-factor, 39-item fitted confirmatory structural equation model presented in Fig. 2 showed adequate factor loadings and indicators/factor pragmatics. Confirmatory factor analyses performed on the latter data-driven factor model of the C-DOGs with respect to the second subsample yielded a six-factor, 36-item fitted confirmatory structural equation model after three iterations of the two-step procedure. In the first iteration, the seven-factor, 40-item fitted confirmatory structural equation model presented one item with a factor loading (*λ* < 0.500) and one factor with fewer than three items. In the second iteration, the six-factor, 37-item fitted confirmatory structural equation model presented one item with a factor loading (*λ* < 0.500). In the third iteration, the six-factor, 36-item fitted confirmatory structural equation model presented in Fig. 3 showed adequate factor loadings and indicators/factor pragmatics. The quality of the adjustment of the theory-driven and data-driven fitted confirmatory structural equation models to the data and their corresponding internal consistency reliability are reported in Table 1. The theory-driven model presented sub-adequate adjustment to the data in light of the TLI (0.894), and the data-driven model presented adequate adjustment to the data in light of all three fit indices considered. The results of the two models are presented in Table 1.

**Table 1.** Theory-driven and data-driven confirmatory structural equation models

Model	*n*	χ^2^	*df*	*p*	CFI	TLI	RMSEA [90% CI]	α	ω
Theory-driven	588	2,831.838	692	<0.001	0.901	0.894	0.073 [0.070, 0.075]	0.690–0.889	0.740–0.955
Data-driven	588	1,792.185	579	<0.001	0.939	0.933	0.060 [0.057, 0.063]	0.610–0.903	0.701–0.934

Model = theory-driven and data-driven confirmatory structural equation models; *n* = model's subsample size; χ^2^ = model's chi-square test chi-square value; *df* = model's chi-square test degrees of freedom value; *p* = model's chi-square test probability value; CFI = model's comparative fit index; TLI = model's Tucker-Lewis fit index; RMSEA [90% CI] = model's root mean square error of approximation along with its corresponding 90% confidence interval; α = model's Cronbach's *α* internal consistency coefficient values range; *ω* = model's McDonald's *ω* internal consistency coefficient values range.

Table 2 reports inter-factor and external correlations.^2^ Overall, we found significant (*p* < 0.05), positive, and moderate (*r* = 0.37) to strong (*r* = 0.70) inter-factor correlations. A similar pattern was found for the correlations between the C-DOG factors and the external variables, with correlations above or equal to *r* = 0.40, bolded in Table 2. Table 3 reports factor loadings of items on each factor and the hypothesized construct of the C-DOG model to which each item originally belonged.

**Table 2.** Inter-factor and external correlations

	F.1	F.2	F.3	F.4	F.5	F.6	IGDT-10	VIS	DASS-D	DASS-A	DASS-S	DERS	Trauma	Sex	Age	H/week
F.1	**1**															
F.2	0.376^**^	**1**														
F.3	**0.456** ^**^	**0.421** ^**^	**1**													
F.4	**0.436** ^**^	**0.474** ^**^	**0.507** ^**^	**1**												
F.5	0.381^**^	**0.530** ^**^	**0.477** ^**^	**0.687** ^**^	**1**											
F.6	**0.404** ^**^	**0.456** ^**^	**0.463** ^**^	**0.707** ^**^	**0**.599^**^	**1**										
IGDT-10	0.381^**^	**0.539** ^**^	**0**.423^**^	**0.552** ^**^	**0.495** ^**^	**0.574** ^**^	**1**									
VIS	0.391^**^	0.399^**^	**0.531** ^**^	**0.460** ^**^	0.362^**^	**0.412** ^**^	0.523^**^	**1**								
DASS-D	0.323^**^	0.337^**^	0.282^**^	**0.521** ^**^	0.379^**^	**0.554** ^**^	0.522^**^	0.274^**^	**1**							
DASS-A	0.235^**^	0.358^**^	0.246^**^	**0.403** ^**^	**0.450** ^**^	0.375^**^	0.444^**^	0.207^**^	0.658^**^	**1**						
DASS-S	0.337^**^	0.372^**^	0.352^**^	**0.420** ^**^	0.362^**^	**0.436** ^**^	0.524^**^	0.271^**^	0.751^**^	0.695^**^	**1**					
DERS	0.312^**^	0.362^**^	0.340^**^	**0.518** ^**^	**0.441** ^**^	**0.504** ^**^	0.528^**^	0.284^**^	0.729^**^	0.628^**^	0.690^**^	**1**				
Trauma	0.240^**^	0.256^**^	0.247^**^	**0.426** ^**^	0.282^**^	**0.410** ^**^	0.352^**^	0.230^**^	0.542^**^	0.415^**^	0.503^**^	0.480^**^	**1**			
Sex	0.043	−0.006	−0.053	0.059^*^	0.017	0.027	−0.037	−0.054	0.100^**^	0.139^**^	0.100^**^	0.082^**^	0.232^**^	**1**		
Age	−0.072^*^	−0.107^**^	−0.136^**^	−0.187^**^	−0.186^**^	−0.170^**^	−0.182^**^	−0.166^**^	−0.099^**^	−0.132^**^	−0.090^**^	−0.164^**^	0.001	0.132^**^	**1**	
H/week	0.159^**^	0.227^**^	0.194^**^	0.239^**^	0.145^**^	0.195^**^	0.241^**^	0.355^**^	0.186^**^	0.110^**^	0.149^**^	0.146^**^	0.173^**^	0.010	−0.053	**1**

** = *p* < 0.01; * = *p* < 0.05; F = factor; IGDT-10 = Internet Gaming Disorder Test; VIS = Videogames Involvement Scale; DASS-D = Depression Anxiety Stress Scale-Depression; DASS-A = Depression Anxiety Stress Scale-Anxiety; DASS-S = Depression Anxiety Stress Scale-Stress; DERS = Difficulties in Emotion Regulation Scale; H = hours. Correlation coefficients for the C-DOGs factors > 0.4 are evidenced in bold.

**Table 3.** Factor loadings of the C-DOG factors

	Item	HD	F.1	F.2	F.3	F.4	F.5	F.6
C-DOGs 18.	I engage in gaming actions that help me approach my negative emotions (e.g. anger) in a more suitable environment	AE	0.83					
C-DOGs 4.	Playing online games helps me to enter a state of peace	GRR	0.67					
C-DOGs 2.	I play online games because it reduces the tension	GRR	0.53					
C-DOGs 41.	After a session of gaming, it is like I “wake up” and suddenly realize that many hours have passed since I started playing	D		0.82				
C-DOGs 8.	I get so immersed in the game that I do not notice the things happening around me in the offline world	BMD		0.69				
C-DOGs 9.	I get so involved in my play that I lose track of time	BMD		0.66				
C-DOGs 23.	I find that overcoming difficult gaming challenges helps me build confidence to deal with life's problems	AE			0.81			
C-DOGs 24.	Through gaming, I feel to develop important skills that help me cope with difficulties in achieving my offline life goals	AE			0.74			
C-DOGs 11.	I have the experience of remembering a past in-game event so vividly that I feel like I am reliving it again	BMD			0.68			
C-DOGs 15.	The perception of some elements of the offline/physical world has changed as a result of places I have visited or experiences I have had in the game (e.g. start noticing grip point on walls after having climbed buildings in a game several times)	BMD			0.62			
C-DOGs 35.	I play online games to avoid feeling judged or rejected by others in offline contexts	E				0.89		
C-DOGs 54.	I find that gaming events are more vivid or memorable than events in my real life	D				0.85		
C-DOGs 34.	I get involved in online games because outside the game world I struggle to express who I really am	E				0.83		
C-DOGs 50.	I need to keep playing because I do not feel safe when I'm not playing	D				0.82		
C-DOGs 36.	I prefer online games rather than face-to-face interactions	E				0.79		
C-DOGs 25.	Expanding my social circles via online gaming prevents me from feeling isolated	AE				0.78		
C-DOGs 38.	I feel that the game is a safer place than the offline world	E				0.77		
C-DOGs 26.	Enjoying the sense of belonging that comes with being a part of a community of gamers (e.g. guilds, teams or general communities) prevents me from feeling lonely	AE				0.75		
C-DOGs 37.	Playing online games allows me to stay away from a “toxic” environment (e.g. family quarrels, school bullying, work problems)	E				0.71		
C-DOGs 14.	I experience bodily sensations of movements as if I were in the videogame when I am not playing	BMD					0.84	
C-DOGs 44.	I feel like my virtual body (avatar) belongs more to me than my body does	D					0.83	
C-DOGs 13.	I can feel as if I am looking at the real world as though I were in the game	BMD					0.79	
C-DOGs 12.	I have the experience of not being sure whether conversations or experiences happened in the game or in offline life	BMD					0.77	
C-DOGs 10.	I find that I can become so involved in the game it feels like it is really happening to me	BMD					0.74	
C-DOGs 42.	During a session of gaming, it happens to me to stop and suddenly realize that I don't recognize the room or the world around me, as if they weren't real	D					0.74	
C-DOGs 39.	I have the experience of feeling as if people, objects and the world around me are not real while this doesn't happen to me while I am playing	D					0.71	
C-DOGs 40.	I sometimes feel like I'm out of my body, watching myself from the outside whilst I am doing something, while this doesn't happen when I'm in the game world	D					0.71	
C-DOGs 53.	Intrusive and disturbing images, thoughts or sounds of the game come to my mind when I am not playing	D					0.68	
C-DOGs 43.	After playing, I feel like my mind has been disconnected from my body	D					0.67	
C-DOGs 48.	I can confuse my own name with that of my character or with my gamertag	D					0.65	
C-DOGs 16.	I engage in specific gaming experiences to “metabolize” (elaborate, process, make sense out of) situations of my offline life that are somehow emotionally similar to those virtual experiences	AE					0.62	
C-DOGs 52.	When I stop playing, disturbing memories and emotions come to my mind	D					0.58	
C-DOGs 31.	I play to protect myself from thoughts and emotions related to future challenges and possible failures in social relationships, at school or in my career	E						0.89
C-DOGs 22.	I find that achieving challenging goals in gaming environments helps me deal with some failures or disappointments in my offline life	AE						0.83
C-DOGs 30.	I keep busy gaming to avoid the difficult challenges of my life rather than deal with them directly	E						0.81
C-DOGs 32.	I keep playing so I can postpone doing something challenging I feel I can't do	E						0.74

C-DOGs = Compensatory-Dissociative Online Gaming scales; GRR = Gaming-Related Relaxation; BMD = Body-Mind Detachment; AE = Active Escapism; E = Escape; D = Dissociation.

Factor 1 refers to a form of emotional displacement (“*I engage in gaming actions that help me approach my negative emotions [e.g., anger, loneliness] in a more suitable environment”)* into the game linked to a sense of peace and relaxation (e.g., “*Playing online games helps me to enter a state of peace*”). Two of the three items of this scale were hypothesized to be Gaming-Related Relaxation items in the C-DOG model. However, given the nature of the first item leading the factor, we labeled it **Emotional Displacement**. This scale showed low yet acceptable internal consistency of Cronbach's *α* = 0.66. Regression analyses showed that ER was significantly (*p* < 0.001) and positively predicted by passion for gaming (*B* = 0.08; *β* = 0.24), the Stress subscale of the DASS (*B* = 0.11; *β* = 0.17), problematic gaming (*B* = 0.10; *β* =0 .12), and Compensation via gaming (*B* = 0.25; *β* = 0.10; *R*^2^ = 0.15 for Step 1, Δ*R*^2^ = 0.23 for Step 4).

Factor 2 describes losing track of time (“*After a session of gaming, it is like I “wake up” and suddenly realize that many hours have passed since I started playing”*) and space (“*I get so immersed in the game that I do not notice the things happening around me in the offline world”*) during gaming that we labeled **Absorption**. This factor showed satisfactory internal consistency of Cronbach's *α* = 0.70. Regression analyses showed that this factor was significantly (*p* < 0.001) and positively predicted by problematic gaming (*B* = 0.28; *β* = 0.35), passion for gaming (*B* = 0.05; *β* = 0.14), the Anxiety subscale of the DASS (*B* = 0.12; *β* = 0.15), and the Goals subscale of the DERS (*B* = 0.16; *β* = 0.17) and negatively predicted by the Strategies subscale of the DERS (*B* = −0.18; *β* = −0.17). In addition, it was significantly (*p* < 0.05) and positively predicted by the hours/week played (*B* = 0.01; *β* = 0.07), by playing RTS games (*B* = 0.60; *β* = 0.06), and by the Clarity subscale of the DERS (*B* = 0.07; *β* = 0.07; *R*^2^ = 0.29 for Step 1, Δ*R*^2^ = 0.35 for Step 8).

Factor 3 describes the perception of increased confidence in managing the challenges of the physical environment (e.g., “*I find that overcoming difficult gaming challenges helps me build confidence to deal with life's problems*”) thanks to some skills developed by gaming (“*Through gaming, I feel to develop important skills that help me cope with difficulties in achieving my offline life goals”*). This effect is reinforced by the particularly vivid and memorable nature of the experiences in the game (“*I have the experience of remembering a past in-game event so vividly that I feel like I am reliving it again*”), such that they can be transferred to the world outside, changing the individual's perception of it (“*The perception of some elements of the offline/physical world has changed as a result of places I have visited or experiences I have had in the game (e.g. start noticing grip point on walls after having climbed buildings in a game several times*)”; Ortiz De Gortari & Diseth, 2022). In accordance with the C-DOG model, we named this factor **Active Escapism** (Giardina et al., 2023, 2024; Kuo et al., 2016). This factor showed good internal consistency of Cronbach's *α* = 0.75. Regression analyses showed that Active Escapism was positively and significantly predicted by passion for gaming (*B* = 0.19; *β* = 0.38; *p* <. 001), the Stress subscale of the DASS (*B* = 0.18; *β* = 0.20; *p* < 0.001), playing MMORPGs (*B* = 0.93; *β* = 0.11; *p* < 0.001), problematic gaming (*B* = 0.09; *β* = 0.08; *p* < 0.05), in-out of the game behavioral consistency (*B* = 0.34; *β* = 0.08; *p* < 0.05), the Nonacceptance subscale of the DERS (*B* = 0.13; *β* = 0.09; *p* < 0.05), and Compensation via gaming (*B* = 0.26; *β* = 0.07; *p* < 0.05). It was negatively predicted by age (*B* = −0.04; *β* = −0.07; *p* < 0.05), sex (*B* = −0.57; *β* = −0.04; *p* < 0.05) and the Depression subscale of the DASS (*B* = −0.90; *β* = −0.09; *p* < 0.05; *R*^2^ = 0.28 for Step 1, Δ*R*^2^ = 0.37 for Step 10).

Factor 4 describes an impairment in relational functioning represented by difficulty in being authentic within offline relationships because of a fear of coming across as inadequate to others (“*I play online games to avoid feeling judged or rejected by others in offline contexts”*) and thus an avoidance of physical interactions and a preference for online interactions and communities, to contain the feeling of loneliness and isolation (e.g., “*Enjoying the sense of belonging that comes with being a part of a community of gamers [e.g. guilds, teams or general communities] prevents me from feeling lonely*”). This dimension may indicate that the social identity of the person is exclusively attached to the game as the only place where they feel free and safe in interacting with others (e.g., “*I need to keep playing because I do not feel safe when I'm not playing*”). For this reason, we named this factor **Virtual Withdrawal**. This factor showed excellent internal consistency of Cronbach's *α* = 0.90. Regression analyses showed that Virtual Withdrawal was significantly (*p* < 0.001) and positively predicted by problematic gaming (*B* = 0.45; *β* = 0.20), Compensation via gaming (*B* = 1.27; *β* = 0.17), the Depression subscale of the DASS (*B* = 0.24; *β* = 0.15), passion for gaming (*B* = 0.19; *β* = 0.19), the Nonacceptance subscale of the DERS (*B* = 0.36; *β* = 0.13), traumatic experiences (*B* = 0.57; *β* = 0.13), and the Clarity subscale of the DERS (*B* = 0.22; *β* = 0.10; *p* < 0.05). It was negatively predicted by in-out of the game behavioral consistency (*B* = −0.77; *β* = −0.09; *p* < 0.001), age (*B* = −0.07; *β* = −0.06; *p* < 0.05) and the Stress subscale of the DASS (*B* = −0.14; *β* = −0.10; *p* < 0.05; *R*^2^ = 0.30 for Step 1, Δ*R*^2^ = 0.49 for Step 10).

Factor 5 describes a level of involvement with the game such that the individual experiences a thinning of the barrier between the virtual and physical worlds, with a consequent confusion in the domains of the body (e.g., “*I feel like my virtual body [avatar] belongs more to me than my body does*”), of perception (e.g., “*I can feel as if I am looking at the real world as though I were in the game*”), of memories (e.g., “*I have the experience of not being sure whether conversations or experiences happened in the game or in offline life”*), and of identity (e.g., “*I can confuse my own name with that of my character or with my gamertag*”). Such involvement that takes on dissociative proportions seems to be linked with altered states of mind (e.g., “*I have the experience of feeling as if people, objects and the world around me are not real while this doesn't happen to me while I am playing*”) and unbearable feelings (e.g., “*When I stop playing, disturbing memories and emotions come to my mind*”) that may be independent of the game and that are kept under control as long as the individual is playing. In this sense, gaming simultaneously generates some dissociative states but alleviates others (e.g., “*I engage in specific gaming experiences to “metabolize” [elaborate, process, make sense out of] situations of my offline life that are somehow emotionally similar to those virtual experiences*”). For this reason, we named this factor **Dissociative Regulation**. It showed excellent internal consistency of Cronbach's *α* = 0.88. Regression analyses showed that Dissociative Regulation was significantly and positively predicted by problematic gaming (*B* = 0.56; *β* = 0.25; *p* < 0.001), the Anxiety subscale of the DASS (*B* = 0.60; *β* = 0.27; *p* < 0.001), Compensation via gaming (*B* = 0.98; *β* = 0.13; *p* < 0.001), passion for gaming (*B* = 0.14; *β* = 0.14; *p* < 0.001), and the Clarity (*B* = 0.26; *β* = 0.10; *p* < 0.05), Control (*B* = 0.40; *β* = 0.12; *p* < 0.001), and Nonacceptance (*B* = 0.22; *β* = 0.09; *p* < 0.05) subscales of the DERS. It was negatively predicted by social gaming (*B* = −1.58; *β* = −0.10; *p* < 0.001), the Stress subscale of the DASS (*B* = −0.26; *β* = −0.14; *p* < 0.001), and the Goals subscale of the DERS (*B* = −0.30; *β* = −0.11; *p* < 0.05; *R*^2^ = 0.24 for Step 1, Δ*R*^2^ = 0.38 for Step 10).

Factor 6 refers to involvement in gaming to avoid the psychic contents (thoughts and emotions) of an expected failure outside the game (“*I play to protect myself from thoughts and emotions related to future challenges and possible failures in social relationships, at school or in my career*”). The feeling underlying such avoidance is being unprepared for the challenges of an uncertain future. In this scenario, the game functions as a mean of avoidance and procrastination (e.g., “*I keep playing so I can postpone doing something challenging I feel I can't do*”), as well as of protection of the Self from the shame of the anticipated failure (e.g., “*I play to protect myself from thoughts and emotions related to future challenges and possible failures in social relationships, at school or in my career*”) and of a boost in self-confidence that helps control the damage (e.g., “*I find that achieving challenging goals in gaming environments helps me deal with some failures or disappointments in my offline life*”). For this reason, we labeled the factor **Failure Escape**. This factor showed excellent internal consistency of Cronbach's *α* = 0.83. Regression analyses showed that Failure Escape was significantly (*p* < 0.001) and positively predicted by problematic gaming (*B* = 0.30; *β* = 0.31), Compensation via gaming (*B* = 0.80; *β* = 0.22), the Depression subscale of the DASS (*B* = 0.18; *β* = 0.23), passion for gaming (*B* = 0.05; *β* = 0.11), the Strategies subscale of the DERS (*B* = 0.14; *β* = 0.10), and traumatic experiences (*B* = 0.20; *β* = 0.09). It was significantly (*p* < 0.05) and negatively predicted by the Stress subscale of the DASS (*B* = −0.08; *β* = −0.09), playing MMORPGs (*B* = −0.42; *β* = −0.05), social gaming (*B* = −0.37; *β* = −0.04), and in-out of the game behavioral consistency (*B* = −0.17; *β* = −0.04; *R*^2^ = 0.33 for Step 1, Δ*R*^2^ = 0.50 for Step 10).

Results of the multiple regression models including the C-DOGs factors as predictors of passion for gaming and problematic gaming are reported in Table 4. When the six factors were examined together as predictors of passion for gaming after we controlled for online game genres, we found significant (*p* < 0.001) and positive effects for Active Escapism (*B* = 0.64; *β* = 0.32), Virtual Withdrawal (*B* = 0.17; *β* = 0.17), Absorption (*B* = 0.38; *β* = 0.13), Emotional Displacement (*B* = 0.28; *β* = 0.10), and Failure Escape (*B* = 15; *β* = 0.07; *p* < 0.05) and a light negative effect for Dissociative Regulation (*B* = −0.08; *β* = −0.08; *p* < 0.05). The model also showed an effect for playing MOBA (*B* = 2.53; *β* = 0.11; *p* < 0.001) and FPS (*B* = 1.19; *β* = 0.11; *p* < 0.001). This model explained 39% of the variance of passion for gaming (*R*^2^ = 0.29 for Step 1, Δ*R*^2^ = 0.39 for Step 8).

**Table 4.** C-DOG scales predicting a passion for gaming and problematic gaming

	C-DOG dimension	Effect (*β*)
Passion for gaming	Active Escapism	0.32^**^
	Absorption	0.17^**^
	Emotional Displacement	0.13^**^
	Failure Escape	0.07^*^
	Dissociative Regulation	−0.08^*^
Problematic gaming	Absorption	0.29^**^
	Failure Escape	0.28^**^
	Virtual Withdrawal	0.18^**^
	Emotional Displacement	0.07^*^

**p < 0.001; *p < 0.05.

When the six factors were examined together as predictors of problematic gaming after we controlled for online game genres, we found significant effects for Failure Escape (*B* = 0.26; *β* = 0.28; *p* < 0.001), Absorption (*B* = 0.36; *β* = 0.29; *p* < 0.001), Virtual Withdrawal (*B* = 0.08; *β* = 0.18; *p <* 0.001), and Emotional Displacement (*B* = 0.10; *β* = 0.07; *p* < 0.05). Active Escapism and Dissociative Regulation were not included in the hierarchical model, as they yielded nonsignificant test values^3^ during the forced entry step. The model also showed an effect for playing MOBA (*B* = 0.49; *β* = 0.05; *p* < 0.05). Step 4 of the hierarchical model that included only the C-DOG scales explained 45% of the variance of problematic gaming (*R*^2^ = 0.33 for Step 1, Δ*R*^2^ = 0.46 for Step 5).

## Discussion

The objective of this study was to develop and validate the Compensatory-Dissociative Online Gaming scales (C-DOGs), a multidimensional assessment measure based on the C-DOG model formulated in our previous work (Giardina et al., 2024). With respect to the five-factor structure hypothesized in our model, our results differed in that they revealed an additional factor and some noteworthy, unexpected outcomes in the content of the scales.

The first factor, which we labeled **Emotional Displacement**, referred to the perception of the game as a more suitable place to which certain emotions (e.g., anger) could be redirected, with an effect of venting and relaxing. Emotional Displacement was weakly yet significantly predicted by problematic gaming, more strongly by a passion for gaming, and moderately by stress. This dimension resembles the adaptive nature of the “coping” subscales identified by existing frameworks of motivations to play (Demetrovics et al., 2011; Király et al., 2022). High scores on this scale indicate that the individual tends to use the gaming environment for the expression of some emotions that would be problematic to express in other contexts, a defense mechanism that psychodynamic literature defines displacement (Di Giuseppe & Perry, 2021).

The second factor referred to the tendency of losing track of time and space when immersed in the game. This dimension represents a specific part of the Body-Mind Detachment construct hypothesized in the C-DOG model (Giardina et al., 2024), which we labeled **Absorption**. Results showed that Absorption was the strongest predictor of problematic gaming among the C-DOG dimensions, and yet it was also found to be associated with intensive but non-problematic involvement in videogames. Absorption was also predicted by anxiety, difficulty in focusing when upset and in recognizing negative emotions, and playing RTS games. Overall, high scores on this scale indicate that when individuals are playing, they detach from the physical world to immerse in the virtual one. Absorption could be sustained by the urge to detach from unrecognized and disturbing emotional states. However, it is important to note that high levels of Absorption could be also found in individuals with an intense yet healthy passion for video games, likely associated with a form of flow (Larche et al., 2021). For example, Jennett et al. (2008) suggested that the degree of perceived immersion in a game is proportionally related to how well the player is doing inside the game, which also relates to the level of state anxiety. In this sense, the slight predictive effect of playing RTS games may be explained by the focus required to simultaneously manage a massive number of variables (e.g., building/development resources, military troops on multiple fronts) in these kinds of games. Similarly to the time spent gaming, Absorption alone is thus a dimension that is poorly discriminative with respect to problematic gaming (Király, Koncz, Griffiths, & Demetrovics, 2023; Király, Tóth, Urbán, Demetrovics, & Maraz, 2017). It should be considered a physiological precursor of a more substantial form of dissociation into the game, such as that identified by the fifth factor, Dissociative Regulation (Guglielmucci et al., 2019).

The third factor was labeled **Active Escapism**. Active Escapism indicates the tendency to “train” certain skills through the vivid experiences and activities made within the game, thus gaining confidence in facing the challenges of the outside world and regulating the negative emotions associated with difficulties in personal achievement (i.e., compensatory function; Boldi et al., 2022; Harley, Pekrun, Taxer, & Gross, 2019; Latinsky & Ueno, 2021). This seems to be especially the case when playing MMORPGs, which offer a wider possibility to simulate experiences in which individuals can prove themselves in front of others. When taken into account with the other C-DOG scales in the multiple regression models, Active Escapism was found to be the best predictor of nonproblematic passion for video games, and its predictive effect with respect to problematic gaming was nonsignificant. In addition, higher levels of Active Escapism were found to be associated with higher stress but lower depression. Overall, high scores on this scale indicate the individuals' tendency to successfully compensate through gaming for their insecurities with respect to performances, which could nonetheless indicate the existence of such insecurities. Active Escapism stresses the simulative nature of gaming, and thus the importance of the concept of simulation for mental health (Giardina et al., 2024). Such results are in line with those of the Gaming in Difficult Life Situations scale by Caro and Popovac (2021), suggesting two subdimensions referring to gaming to simulate offline challenges in an effort to cope (*simulation*) and gaming to feel a sense of achievement when dealing with difficulties (*sense of purpose*). Our results also align with those of Bender and Gentile (2020) who found positive correlations between satisfaction of basic psychological needs for autonomy and competence within the game and in life in general.

The fourth factor, which we labeled **Virtual Withdrawal**, describes an impairment in relational functioning based on a strong feeling of inadequacy that the individual tries to overcome by avoiding social interactions in the physical world and investing in the more social aspects of the game. This scale was the most strongly predicted by experiences of psychological trauma, and it was strongly predicted by depression, difficulty in describing and accepting negative emotions (i.e., tendencies to feel ashamed or guilty when upset), the tendency to compensate via gaming and to behave in the game in a way that is inconsistent with the behavior outside of it. Virtual Withdrawal has relevant implications for mental health. High scores on this scale indicate an aptitude to withdraw into virtual sociality, perceived as safer and more controllable, to counteract isolation from sociality in the physical world, which is instead perceived as unpredictable and threatening. This perception seems to be due to a fragile sense of self developed from adverse relational experiences such as bullying at school, or other experiences of rejection and social exclusion. Therefore, the high attachment to gaming mixing problematic and passionate features of individuals scoring high on this scale depends on the need to feel accepted by a community of their choice and mitigate the sense of loneliness that would otherwise permeate their everyday life (Heng, Zhao, & Wang, 2021; Infanti, Valls-Serrano, Perales, Vögele, & Billieux, 2023; Kowert, Domahidi, Festl, & Quandt, 2014). These results are in line with past studies highlighting that the mediated appearance through the avatar and the possibility to easily interrupt interactions within the game promote a perception of safety and greater control over social interactions in socially anxious individuals (Green, Delfabbro, & King, 2021; Sioni, Burleson, & Bekerian, 2017; Szolin et al., 2022). Accordingly, Virtual Withdrawal may be linked to the *Hikikomori* phenomenon (Bonnaire & Roignot, 2022; Dell’Osso et al., 2023). *Hikikomori* is a term referring to a specific kind of social withdrawal by adolescents and young adults that was initially acknowledged in Japan and is now being used to explain an increasing number of social withdrawals with similar characteristics and onset in Europe (Kato, Shinfuku, & Tateno, 2020). We posit the hypothesis that the experiences captured by Virtual Withdrawal are representative of a portion of socially withdrawn individuals who depend on gaming to stay connected with the world outside their room. In this sense, Virtual Withdrawal may constitute the link between problematic gaming and *Hikikomori* (Giardina et al., 2024; Stavropoulos et al., 2019).

The fifth factor, **Dissociative Regulation**, refers to an extreme level of immersion in online games that simultaneously alleviates existing dissociative states and produces new ones. Dissociative Regulation was the only C-DOG scale that negatively predicted passion for gaming, and it did not predict problematic gaming. However, Dissociative Regulation was also strongly and positively predicted by problematic gaming, as well as by anxiety and difficulties in emotion regulation, with specific reference to the struggles in describing, controlling, and accepting negative emotions.

Overall, these results suggest that individuals scoring high on this dimension may experience overwhelming anxiety with dissociative states that they try to mitigate by gaming, with the “boomerang effect” of a further disconnection from the physical world and a fusional identification with the virtual one. In this sense, this condition may be linked to detachment from the physical body and strong emotional attachment to its virtual representation (e.g., “*I feel like my virtual body (avatar) belongs more to me than my body does*”), as if the character played is more easily identifiable as the “true self” (Winnicott, 2018). Future studies should explore the association of this process with different kinds of avatar-gamer relationships and perceptions of the avatar, including when it is perceived in a social way as an adventure's companion (Banks & Bowman, 2016, 2021; Casale, Fioravanti, & Musicò, 2022, 2023).

Interestingly, this dimension was also strongly predicted by the tendency to game as a lone player, lower stress, and lower difficulty in staying focused when experiencing negative emotions. Such a pattern recalls the “Hardcore Gamers” identified in a previous study by Billieux et al. (2015), a subgroup of gamers presenting with problematic escapism and achievement-oriented gameplay while being characterized by good levels of self-esteem, premeditation of their actions, and perseverance in reaching their goals. Billieux et al. (2015) hypothesized that this profile comprises potentially obsessive-compulsive-prone individuals who may perform very well and be highly ranked inside the game – thus boosting gaming-dependent self-esteem – but who are incapacitated in implementing the same attitude outside the game.

The sixth and last factor refers to a specific form of avoidance via gaming linked with the fear of failing, which we labeled **Failure Escape**. This dimension was the most strongly associated with problematic gaming, both as predictor and outcome, after Absorption. Furthermore, it was strongly predicted by depression, experiences of psychological trauma, and the feeling of having no effective strategies to deal with negative emotions. In accordance with the hypothesized Escape dimension of the C-DOG model, high Failure Escape scores suggest that individuals may have internalized a feeling of powerlessness, resignation, and lack of self-efficacy that they problematically try to alleviate by proving themselves in the game while avoiding the original problem. In other words, the individual may feel burdened by tasks they cannot accomplish, in a more general fear of not being up to what the future will bring. This feeling may be linked to past experiences of frustration of psychological needs in both physical and virtual environments, which would justify the feeling of resignment and the depressed mood (Allen & Anderson, 2018; Tóth-Király et al., 2019). Accordingly, gaming habits associated with Failure Escape are inconsistent with respect to those outside of the game, with a tendency to play alone and being less likely to play MMORPGs, a game genre with important social component offering complex worlds and a high degree of freedom. We suggest that this specific type of avoidance can be found among individuals for whom overly high expectations of one's performance rebound on a devalued self, which may seek to be redeemed, or at least relieved, by feeling competent and valuable within the game (Di Blasi et al., 2020; Forrest, King, & Delfabbro, 2017; Zsila et al., 2023). For example, it may be the case for individuals who internalized higher parental criticism and tend to develop perfectionism tendencies and thus over-engage with failure in gaming (Zsila et al., 2023). Another example could be vulnerable narcissistic individuals (but not grandiose ones), showing greater difficulties in emotion regulation, which more likely leads them to escape into the game problematically (Di Blasi et al., 2020). This finding also echoes anthropological investigations of gamers highlighting the importance in this subculture of “playing well” and the adverse outcomes of not being able to feel like one is doing so (Jennett et al., 2008; Snodgrass et al., 2017). In this sense, Failure Escape and Active Escapism would represent two different potential reactions to failure: disengaged and constructive, respectively (Zsila et al., 2023).

### Passion for gaming and problematic gaming according to the C-DOGs

The C-DOG dimensions contributed to better delineating the difference between an intensive, passionate, and yet healthy involvement in videogames versus problematic gaming (Billieux et al., 2019; Razum & Huić, 2023; Snodgrass, Dengah, Polzer, & Else, 2019). Results showed that Active Escapism had the strongest predictive effect on passion for gaming, followed by Absorption, Emotional Displacement, and Dissociative Regulation. Regarding problematic gaming, Absorption and Failure Escape were almost equally predictive, followed by Virtual Withdrawal and Emotional Displacement. Therefore, an intense passion for gaming is associated with a mix of concentration toward the game, a tendency to gain a sense of relaxation by channeling negative emotions into it, and compensating for the lack of a sense of mastery in the physical world by being good at the game and learning from it. On the other hand, problematic gaming relates to stronger absorption in the game and the tendency to withdraw into the virtual environment because of the negative emotions linked with failure and with adverse relational experiences.

### Comparison with the theoretical model

Although the C-DOGs confirm some of the constructs proposed in the C-DOG model, it also presents some noteworthy differences. The most evident is the additional Virtual Withdrawal factor, which was not hypothesized in the theoretical model. The formation of this factor, loading avoidant and compensatory items around the content of social struggles, stresses the importance of these struggles in individuals with problematic gaming. Specifically, it suggests that social anxiety (Gioia, Colella, & Boursier, 2022), avoidant and vulnerable narcissistic personality traits (Di Blasi et al., 2020; Gervasi et al., 2017), and all psychological conditions that include a strong sense of inadequacy with consequent social isolation (Dell’Osso et al., 2023; Stavropoulos et al., 2019) are central sustaining factors of problematic gaming symptoms (Király et al., 2023).

Another difference worth mentioning is that the items generated for Active Escapism to capture the compensatory movement toward the game are spread across all C-DOG dimensions, except for Absorption. Despite the Active Escapism dimension found reflects the existence of a specific and relatively adaptive compensatory construct, these results suggest that simulation via gaming may serve a cross-cutting compensatory function. The nature of this compensation seems to change depending on what the individual is struggling with: social compensation in the case of Virtual Withdrawal, emotional processing in the case of Dissociative Regulation, and competence compensation in the case of Failure Escape. In terms of the overall theoretical model, this means that the compensatory-dissociative continuum itself was not confirmed. Moreover, unlike the hypotheses of the C-DOG model, our results suggest that Failure Escape and Absorption predict problematic gaming more strongly than Dissociative Regulation does. However, establishing the most problematic C-DOG dimension may require broader considerations. Indeed, considering the predictors of the C-DOG scales, Dissociative Regulation and Virtual Withdrawal may imply greater psychological distress and impairments overall. For example, Failure Escape showed the lowest number of significant predictors among emotion regulation difficulties and a weaker association with emotional distress than Dissociative Regulation did. Furthermore, Dissociative Regulation was the C-DOG scale most strongly predicted by problematic gaming, anxiety, and all emotion regulation difficulties. Similarly, Virtual Withdrawal showed very strong associations with traumatic experiences. A final noteworthy difference from the theoretical model is that the hypothesized Body-Mind Detachment and Dissociation constructs of the C-DOG model, mainly representing a normal and maladaptive form of dissociation, respectively, were both subsumed by Dissociative Regulation. This finding reinforces the view that dissociation is a unique dimensional process (Guglielmucci et al., 2019).

### Originality of the C-DOGs

Considering that several items were collected or inspired from existing frameworks, the C-DOGs share similarities with these models. For example, Active Escapism recalls the self-expansion positive dimension of escapism hypothesized by Stenseng, Rise, and Kraft (2012, 2021), the skill development motivation to play identified by Demetrovics et al. (2011), or the compensation dimension of the User-Avatar Bond Scale (Blinka, 2008; Blinka, Siřínková, & Stašek, 2023). However, Active Escapism is the first dimension that directly investigates not only how playing the game enriches the individual, but also what the individual is compensating for through the game (i.e., a struggle around autonomy and competence psychological needs) and how. Failure Escape can also be traced to the negative escapism facets of previous literature and, more generally, to avoidant coping (Demetrovics et al., 2011; Hagström & Kaldo, 2014; Melodia et al., 2022). However, Failure Escape identified the fear of being unable to deal with future life challenges and failing as a specific form of avoidance strongly linked with problematic gaming, which is fundamental in clinical settings for youth (Shumaker & Manning, 2022). Regarding Dissociative Regulation, it contains some dissociative features that have also been referred to as game transfer phenomena (Ortiz De Gortari & Diseth, 2022). These phenomena include the sensation of movement while not playing, body distortion in relation to the avatar, or mix-ups between physical and virtual worlds. However, the C-DOGs elaborate these experiences in the context of clinical treatment and evaluation and thus in relation to psychopathological processes. In this sense, dissociation is a fundamental process of the mind on par with emotional dysregulation or attachment styles (American Psychiatric Association, 2022; Blasi et al., 2019; Casale et al., 2022; Guglielmucci et al., 2019; Santoro et al., 2021). With respect to Virtual Withdrawal, to the authors' knowledge, this dimension is unprecedented. Overall, the C-DOGs is a useful tool to use in clinical settings for case formulation, as well as for tracking changes in patients' relationship with gaming across therapy sessions.

## Limitations

This study has some noteworthy limitations. First, we used a problematic gaming measure (IGDT-10; Király et al., 2017) that refers to the addiction framework proposed in the *DSM-5 *(American Psychiatric Association, 2022). Several study authors have pointed out the limitations of using this framework to measure problematic gaming (Castro-Calvo et al., 2021; Charlton & Danforth, 2010; Infanti et al., 2023; Razum, Baumgartner, & Glavak-Tkalić, 2023), and future work should test the C-DOGs associations with gaming disorder measures based on *ICD-11* criteria (World Health Organization, 2018). With respect to the Italian adaptation of the Videogames Involvement Scale (Snodgrass et al., 2017) that was translated for the purpose of the present study, two out of the three fit indices transparently reported were sub-adequate relative to the thresholds we determined for adequate adjustment to the data. Therefore, results involving this adaptation should be interpreted with some precaution. In addition, this study was conducted on a community sample with a cross-sectional design. Given the clinical relevance of the dimensions identified, future studies should test the C-DOGs in clinical samples and evaluate the reliability of the scales with longitudinal designs. In a similar vein, future studies should investigate whether recurrent sets of C-DOG dimensions can be found among problem gamers to further facilitate clinical case formulation.

## Conclusions

In the context of our critical review of the role of escapism among compensatory and dissociative processes associated with problematic gaming, we formulated the C-DOG model (Giardina et al., 2024). In this study, we developed and tested a new multidimensional assessment measure that was based on this model. The dimensions identified showed distinctive characteristics and patterns of associations: Emotional Displacement is a form of relaxation coming from the redirection of negative emotion into the game; Absorption represents detachment of the player from time and space due to immersion in gaming; Active Escapism is vivid engagement in the game to boost self-confidence in order to reach physical life objectives; Dissociative Regulation is a highly problematic level of engagement meant to process unbearable anxiety; Virtual Withdrawal is a maladaptive solution to balance impaired social functioning that is associated with traumatic experiences and pervasive depression; and finally, Failure Escape is a form of avoidance via gaming specific to the fear of future failures. Besides shedding light on the debate about the concept of escapism, clinicians and researchers in future studies can use the subscales of the C-DOGs separately or in combination to examine the dynamics of the central processes that sustain problematic gaming in more depth, thus orienting treatment and opening new lines of understanding for research in the field. Specifically, future studies should use the C-DOG dimensions to better understand the role of the avatar-gamer relationship in problematic gaming and the link between *Hikikomori* conditions and problematic gaming, and to test their different associations with motivations to play, as well as with the satisfaction and frustration of basic psychological needs. Furthermore, future studies should validate the scale in other languages, starting with English, and test its clinical utility and other practical applications. Overall, the C-DOGs is the first assessment tool to explore the development of forms of escapism, avoidance, relaxation, and dissociation in a manner that is specific to engagement in gaming in the context of the same model.

## Supplementary materials

Supplementary materials in both Italian and English languages, including the list of measures used, the outputs of the regression analyses, the full pool of 54 candidate items with references, the final version of the C-DOGs with handy indication for clinical administration and interpretation of the scales, and the scoring sheets for clinical use are available at https://osf.io/vgrwk/. The scale and the scoring can also be found in the Appendix.

## Footnotes

1 For a comprehensive discussion about how individuals can compensate with escapism via gaming, please see Giardina et al. (2024).

2 The complete correlation matrix, including the DERS subscales, is available at https://osf.io/vgrwk/.

3 Post hoc forward hierarchical regression analyses showed that the significant effect of Dissociative Regulation on Internet Gaming Disorder disappeared after Absorption was introduced into the model.

## APPENDIX

### Compensatory-Dissociative Online Gaming Scales (C-DOGs): theoretical background

The function of this tool is to support the clinical assessment of individuals who seek help-or are referred-for suspected video game addiction. The tool allows the investigation of the processes of compensation, dissociation, escapism and escape, which are particularly relevant to understanding the individual's relationship with problematic gaming. The Compensatory-Dissociative Online Gaming (C-DOG; Giardina et al., 2024) model underlying the tool views video games as a virtual environment, part of the everyday reality of many individuals, that can be integrated to varying degrees with their physical world. This environment has the characteristic, due in part to its non-physicality, of allowing the simulation of experiences and in particular the satisfaction of basic psychological needs that might not be met in the physical world, facilitating the regulation of respective affective states. Given this simulative nature, video games would have an emotional processing function similar to that of dreams.

In the C-DOG model, the different degree of integration between the virtual environment of the game and the physical environment, with their respective parts of the self, determines a continuum between compensatory (less problematic) and dissociative (more problematic) involvement in the video game. Specifically, on the compensatory side there is a homeostatic relationship between the two environments, in which the individual experiencing deficiencies in his or her basic psychological needs makes use of the simulative environment of the game to enrich his or her life in the physical world. At an intermediate position on the continuum, we have the escape processes that arise from the rejection of the physical environment that leads to taking refuge in the virtual one without actively taking advantage of it. On the dissociative side, the two environments and the respective parts of the self are disconnected. The measurement scales presented below operationalize the psychological processes that have been empirically derived from this theoretical model.

### Indication for use

The C-DOGS has no cut-offs for the time being. It can be used to feedback the patient or the family about the prevailing processes that characterize his or her relationship with gaming, decide how to approach his or her symptom, and evaluate the course of the psychotherapy. The scales should be interpreted as follows.

### Emotional Displacement (ED)

This dimension refers to the redirection of certain negative emotions (such as anger) to gaming, with an effect of venting and relaxing. This dimension is loosely related to problem gaming and more related to intensive and passionate, yet not problematic, involvement in video games. High scores on this scale indicate that the individual tends to use the gaming environment for the expression of some emotions that would be problematic to express in other contexts, a mature defensive mechanism that in psychodynamic terms is called displacement (Di Giuseppe & Perry, 2021).

### Absorption (A)

This dimension refers to the tendency to lose track of time and space while playing. Absorption was found to be the strongest among the other dimensions of the C-DOG scale in predicting problem gaming, and yet it was also found to be associated with intensive but non-problematic involvement in videogames. Absorption is also associated with anxious states, difficulty staying focused when upset, and recognizing negative emotions. Overall, high scores on this scale indicate that when the individual is playing, he or she detaches from the physical world to immerse in the virtual one to distract him or herself from unrecognized and disturbing emotional states. However, it is also important to note that high levels of Absorption could be found in individuals with a passion for video games and thus be associated with a form of flow, thus resulting in a dimension that is poorly discriminative with respect to problematic gaming. It should be considered a precursor to a more severe form of dissociation, as in the below described Dissociative Regulation dimension.

### Active Escapism (AE)

Active Escapism indicates the tendency to make use of the simulative nature of the game and the vivid experiences made within it to “train” with respect to certain skills, thus helping the individual gain confidence in facing the challenges of the outside world and to regulate the negative emotions associated with difficulties in personal achievement. Compared with the other scales, Active Escapism was found to be the most associated with nonproblematic passion for video games, and its association with problematic gaming was nonsignificant. In addition, higher levels of Active Escapism were found to be associated with higher levels of stress but lower levels of depression. Overall, high scores on this scale indicate the individual's tendency to successfully compensate through gaming for his or her insecurities with respect to performance, which could nonetheless indicate the existence of such insecurities.

### Virtual Withdrawal (VW)

The Virtual Withdrawal dimension describes an impairment in social and relational functioning based on strong feelings of inadequacy, which the individual tries to cope with by avoiding interactions in the physical world and investing in the more social aspects of gaming. This dimension was the most associated with experiences of psychological trauma, but it was also found to be associated with depression, difficulty describing and accepting negative emotions (i.e., a tendency to feel ashamed or guilty when upset), and a tendency to behave differently in the game than outside of it. High scores on this scale indicate an aptitude to withdraw into virtual sociality, perceived as safer and more controllable, to counteract isolation from sociality in the physical world, which is instead perceived as unpredictable and threatening due to a fragile sense of self developed from adverse relational experiences such as school bullying, or other experiences of rejection and social exclusion. Thus, the attachment to gaming of individuals with high scores on this scale, which combines problematic but also passionate elements, might depend on the need to feel accepted by a community of their choice and thus mitigate the sense of loneliness and isolation that would otherwise permeate their daily lives.

### Dissociative Regulation (DR)

Dissociative Regulation refers to an extreme level of immersion in the game that simultaneously alleviates existing dissociative states and produces new ones. It can thus be considered a dysfunctional strategy for regulating dissociative states. Indeed, at this level of immersion the individual experiences a thinning of the barrier between the physical and virtual worlds, resulting in confusions in body perception, general perception, memory, and identity. However, this involvement that takes on dissociative proportions seems to be related to altered states of mind and overwhelming emotions that are independent of the game and are kept in check as long as the individual continues to play. Intensive, unproblematic involvement in video games might play a protective role with respect to these experiences, which seem instead to be related to very marked anxiety states and emotional dysregulation, with particular reference to difficulty in describing, governing, and accepting negative emotions. Overall, high scores on this scale suggest that the individual might experience overwhelming anxiety with dissociative states that are mitigated by gaming, with the boomerang effect of further disconnection from the physical world and a fusional identification with the virtual one.

### Failure Escape (FE)

Failure Escape refers to a specific form of avoidance via gaming linked to the need to ward off the psychic contents (thoughts and emotions) of a feared and expected future failure. Faced with the feeling of being unprepared for the challenges of an uncertain future, the game thus functions both as a means of avoidance and procrastination and as protection of the self from the shame of a mentally anticipated failure. Compared to the other dimensions, Failure Escape was found to be the most strongly associated with problematic gaming, after Absorption. It is also strongly predicted by depression, traumatic experiences, and perceived lack of effective strategies to deal with negative emotions. High scores on this scale suggest that the individual may have interiorized feelings of helplessness and resignation, which he or she attempts to alleviate through gaming while avoiding the original problem. These feelings could be related to experiences of frustration of basic psychological needs, whether in the physical or virtual environment, which would account for the feelings of resignation and the depressed mood.

### Compensatory-Dissociative Online Gaming scales (C-DOGs)

Please use the following scale of values to indicate how representative each of the statements is of your experience with online gaming during the last year (last 12 months). There are no good or bad statements. Try to be as transparent as possible regarding your experience.

**Table:**

**1** **Not at all representative**	**2** **Not very representative**	**3** **Not a little nor very representative**	**4** **Very representative**	**5** **Fully representative**

		1	2	3	4	5
1.	I get so immersed in the game that I do not notice the things happening around me in the offline world					
2.	I play online games because it reduces the tension					
3.	I find that I can become so involved in the game it feels like it is really happening to me					
4.	I have the experience of not being sure whether conversations or experiences happened in the game or in offline life					
5.	I have the experience of remembering a past in-game event so vividly that I feel like I am reliving it again					
6.	Expanding my social circles via online gaming prevents me from feeling isolated					
7.	I find that achieving challenging goals in gaming environments helps me deal with some failures or disappointments in my offline life					
8.	I get so involved in my play that I lose track of time					
9.	Playing online games helps me to enter a state of peace					
10.	I can feel as if I am looking at the real world as though I were in the game					
11.	I experience bodily sensations of movements as if I were in the videogame when I am not playing					
12.	The perception of some elements of the offline/physical world has changed as a result of places I have visited or experiences I have had in the game (e.g. start noticing grip point on walls after having climbed buildings in a game for several times)					
13.	Enjoying the sense of belonging that comes with being a part of a community of gamers (e.g. guilds, teams or general communities) prevents me from feeling lonely					
14.	I keep busy gaming to avoid the difficult challenges of my life rather than deal with them directly					
15.	After a session of gaming, it is like I “wake up” and suddenly realize that many hours have passed since I started playing					
16.	I engage in gaming actions that help me approach my negative emotions (e.g. anger) in a more suitable environment					
17.	I engage in specific gaming experiences to “metabolize” (elaborate, process, make sense out of) situations of my offline life that are somehow emotionally similar to those virtual experiences					
18.	I have the experience of feeling as if people, objects and the world around me are not real while this doesn’t happen to me while I am playing					
19.	I find that overcoming difficult gaming challenges helps me build confidence to deal with life's problems					
20.	I get involved in online games because outside the game world I struggle to express who I really am					
21.	I play to protect myself from thoughts and emotions related to future challenges and possible failures in social relationships, at school or in my career					
22.	I sometimes feel like I'm out of my body, watching myself from the outside whilst I am doing something, while this doesn't happen when I'm in the game world					
23.	During a session of gaming, it happens to me to stop and suddenly realize that I don't recognize the room or the world around me, as if they weren't real					
24.	Through gaming, I feel to develop important skills that help me cope with difficulties in achieving my offline life goals					
25.	I play online games to avoid feeling judged or rejected by others in offline contexts					
26.	I keep playing so I can postpone doing something challenging I feel I can’t do					
27.	After playing, I feel like my mind has been disconnected from my body					
28.	I feel like my virtual body (avatar) belongs more to me than my body does					
29.	I prefer online games rather than face-to-face interactions					
30.	I can confuse my own name with that of my character or with my gamertag					
31.	Playing online games allows me to stay away from a “toxic” environment (e.g. family quarrels, school bullying, work problems)					
32.	When I stop playing, disturbing memories and emotions come to my mind					
33.	I feel that the game is a safer place than the offline world					
34.	Intrusive and disturbing images, thoughts or sounds of the game come to my mind when I am not playing					
35.	I find that gaming events are more vivid or memorable than events in my real life					
36.	I need to keep playing because I do not feel safe when I’m not playing					

### Scoring

**Emotional Displacement (ED)** = 2, 9, 16

**Absorption (A)** = 1, 8, 15

**Active Escapism (AE)** = 5, 12, 19, 24

**Dissociative Regulation (DR)** = 3, 4, 10, 11, 17, 18, 22, 23, 27, 28, 30, 32, 34

**Virtual Withdrawal (VW)** = 6, 13, 20, 25, 29, 31, 33, 35, 36

**Failure Escape (FE)** = 7, 14, 21, 26

The C-DOGS has no total score and no reverse items. The score of the single scales can be obtained by summing the scores of the respective items. Please refer to the scoring Excel sheet available at https://osf.io/vgrwk/ to speed up the process.
